# Kinematic Alignment Bi-unicompartmental Knee Arthroplasty With Oxford Partial Knees: A Technical Note

**DOI:** 10.7759/cureus.28556

**Published:** 2022-08-29

**Authors:** Takafumi Hiranaka, Takaaki Fujishiro, Motoki Koide, Koji Okamoto

**Affiliations:** 1 Department of Orthopaedic Surgery and Joint Surgery Centre, Takatsuki General Hospital, Takatsuki, JPN

**Keywords:** bi-unicompartmental, unicompartmental, arthroplasty, technique, surgery, knee

## Abstract

Bi-unicompartmental knee arthroplasty (BiUKA) is an alternative to total knee arthroplasty for selected patients. Although it is thought to be technically demanding, the technique has not been previously described in detail. Kinematic alignment (KA) implantation and bone cuts parallel *to* the native joint line would be beneficial to ensure optimal mechanical loading. Here, we detail a technique for KA-BiUKA using the Oxford partial knees. The joint line is identified using the spoon of the microplasty instrumentation system with/without the accessory spoons. The tibia is cut parallel with the joint line using a side-slidable ankle yoke so that the inclination of the cutting block is parallel with the spoon surface. After defining the horizontal bone-cutting lines, the predominantly affected condyle is operated upon, followed by the lesser affected condyle. Although custom-made devices are required, the technique is useful and reproducible in the performance of KA-BiUKA with the Oxford partial knees.

## Introduction

Unicompartmental knee arthroplasty (UKA) is an attractive surgery for unicompartmental knee osteoarthritis with functioning anterior cruciate ligament (ACL) [1,2]. It is characterized by quicker recovery, fewer systemic complications, lower postoperative mortality, and better range of motion than total knee arthroplasty (TKA) [3-5]. Another advantage of UKA is the retention of the ACL; once the ACL is sacrificed to facilitate a TKA, minor instability and alteration of kinematics are inevitable [6]. Unlike TKA, the original kinematics and joint stability can be retained in UKA, with improved patient satisfaction [7-9].

Despite these benefits, the usage of UKA depends on the integrity of the lateral compartment cartilage [10]. If the lateral compartment is damaged, conversion to TKA is unavoidable, even if the ACL is healthy. Bi-cruciate retaining (BCR) TKA is a possible alternative to conventional TKA, but it is a technically demanding procedure, and the results are not always consistent [6,11]. As described in the four-bar linkage theory, the ligament condition and morphology perfectly correspond to each other. If the morphology of the component matches the native morphology, the results can be excellent; otherwise, tightness and looseness inevitably emerge at certain angles. Complete replication of both medial and lateral components using existing TKA components is thus virtually impossible.

Bi-compartmental knee arthroplasty (BiUKA) is a potentially useful alternative to BCR because both compartments can be resurfaced individually [12-14]. Moreover, the kinematic alignment (KA) procedure is also possible if the components align with the original coronal joint line (CJL) obliquity. Performing KA-BiUKA with Oxford partial knees (OPKs) is also beneficial because the femoral components of OPK are partly spherical, meaning it can serve as a good imitation of the cylindrical axis. Moreover, bone cuts implemented along the CJL might be advantageous for the mechanical properties. Despite such benefits, however, there are no previous reports on BiUKA using OPKs (BiOUKA) except for its initial stage [15], where the surgical technique and instruments are immature and staged BiUKA for lateral compartment osteoarthritis after medial UKA [16]. We have modified the microplasty instruments to ensure tibial cuts parallel to the joint line. And there are no reports of KA-BiOUKA using additional components. This technical note describes KA-BiOUKA using custom-made instruments in detail.

## Technical report

Patient selection

The indication of KA-BiOUKA is functioning ACL and cartilage damage in both medial and lateral compartments. Full-thickness cartilage defects should be found in at least one compartment. In most cases, BiOUKA is a conversion from medial or lateral OPK owing to the intraoperative finding of cartilage damage on the opposite femoral condyle. BiOUKA is not applicable for severe patellofemoral joint diseases, such as bone defects and subluxation.

Preoperative radiographical planning

**Figure 1:**
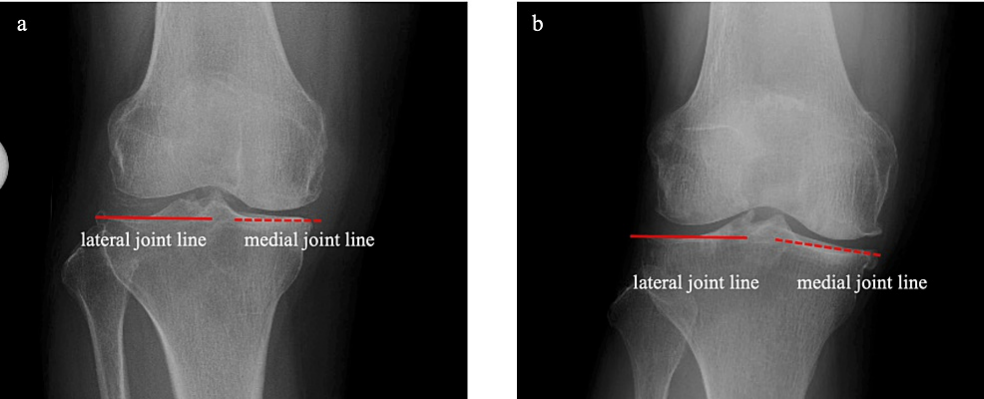
Preoperative radiographic classification (a) Leveled type: The medial and lateral tibial surfaces are aligned. In this type, the medial tibial surface represents the CJL obliquity. (b) Uneven type: The two tibial surfaces are not aligned. The posterior condylar axis is used to define the CJL obliquity. CJL: Coronal joint line.

Preoperative anteroposterior radiography is used for planning. The medial and lateral joint lines are identified as the tangential line of the tibial articular surfaces. If both lines are straight and on the same level (leveled type; Figure 1, Panel a), the medial or lateral joint line is considered to be the CJL, and the single-spoon technique is used (described below). Otherwise, in the case of uneven type (Figure 1, Panel b), the double-spoon technique is performed with reference to the posterior condylar axis (PCA) intraoperatively. The predominantly affected condyle is determined, and this is operated first.

Joint opening

**Figure 2:**
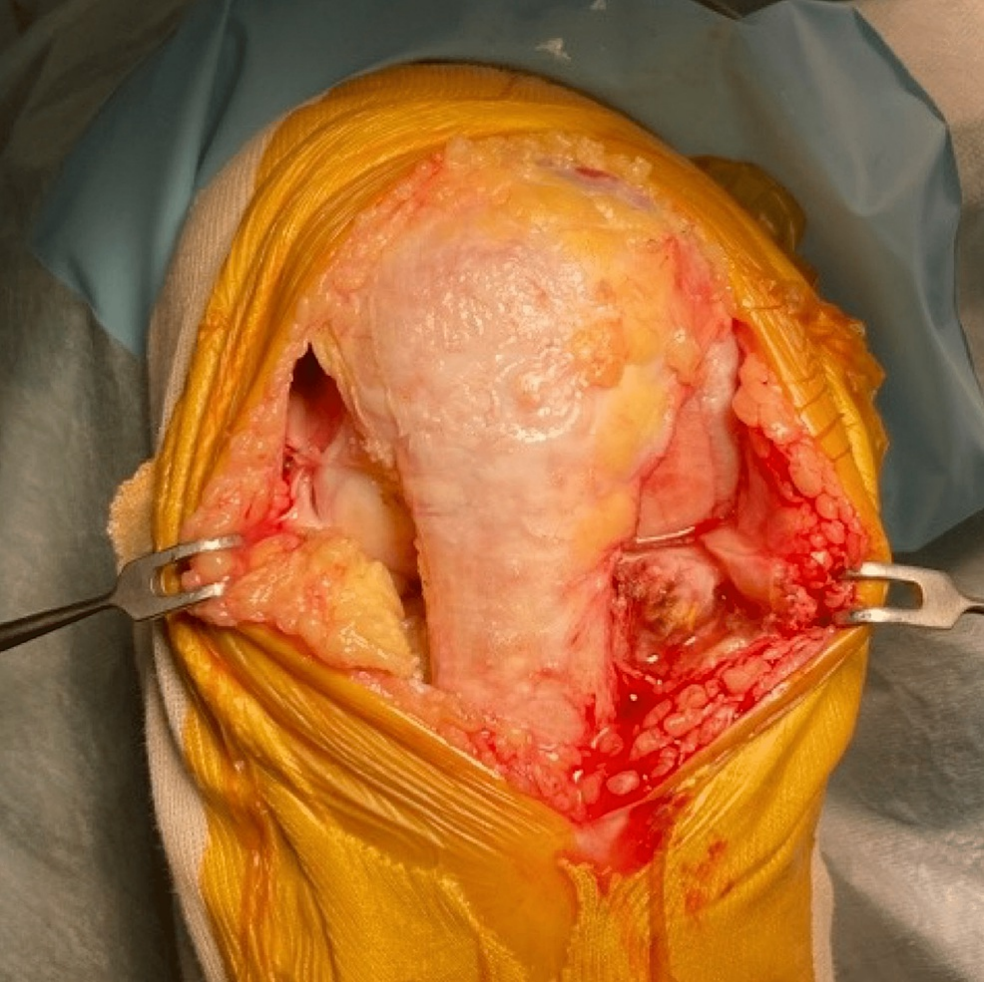
Joint opening For the medial OA, the medial parapatellar skin incision is used. The medial capsulotomy is implemented, and if lateral cartilage damage is found, the skin incision is extended proximally and slightly laterally. The skin flap is then peeled laterally to facilitate lateral capsulotomy. OA: Osteoarthritis.

The medial parapatellar incision and medial parapatellar capsulotomy are performed for medial osteoarthritis (OA). Once the lateral cartilage lesion is found, the skin is peeled laterally so that the lateral border of the patella and patellar tendon is exposed. A lateral parapatellar capsulotomy is then added so that the lateral compartment can be manipulated (Figure 2). For the lateral OA, the lateral parapatellar approach is made and skin is peeled medially to expose the medial border of the patella and patella tendon, and this is followed by medial capsulotomy. Oxford mobile-bearing UKA is used for the medial side and fixed-lateral Oxford (FLO) is used for the lateral side. With the exception of the above-mentioned decision process regarding the tibial cutting plane, both procedures are implemented following the manufacturer-provided operation manuals [17].

Deciding the tibial cutting plane

**Figure 3:**
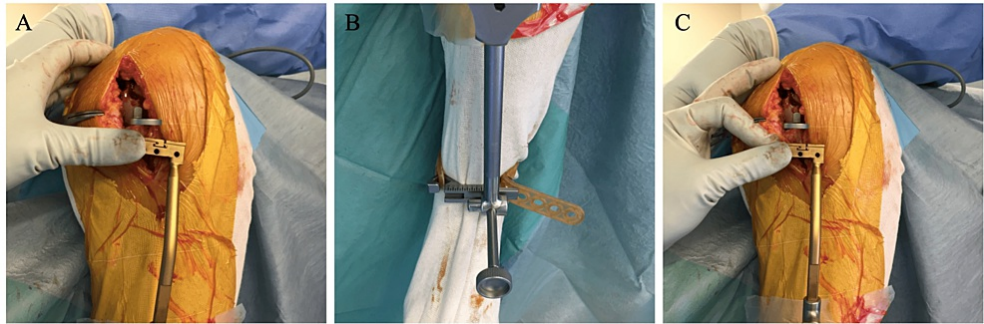
The medial spoon technique for the cutting line definition (A) A spoon is inserted into the medial joint space. In straight-type knees, the inclination of the spoon represents the CJL inclination, the target of the cutting line. (B) The inclination of the tibial cutting block is different from that of the spoon. (C) The slide bar of the ankle yoke is adjusted so that the spoon and the cutting block are parallel. CJL: Coronal joint line.

After joint opening and osteophyte removal, the tibial cutting plane is set parallel with the CJL. The single-spoon technique is used for leveled-type knees. The spoon gauge is inserted into the dominantly affected condyle, representing joint line inclination (Figure 3, Panel A). Our custom-made side-slidable ankle yoke is connected to the extramedullary (EM) rod instead of the original ankle yoke [18]. The sagittal inclination of the EM rod is adjusted so that it is parallel with the anterior cortex of the tibia. The cutting block is set just below the spoon. In most cases, the spoon is not parallel but rather varus to the cutting block (Figure 3, Panel B). The custom-made yoke is then slid laterally until the cutting block and the spoon are parallel (Figure 3, Panel C).

**Figure 4:**
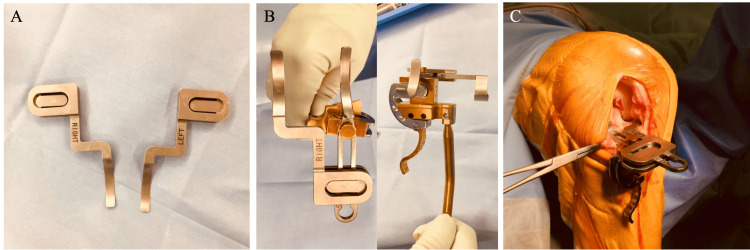
The double-spoon technique (A) Accessory spoons. The accessory spoons are spoons of 0.5 mm thickness that can be joined with conventional spoons. (B) Once the accessory spoons are incorporated with the conventional spoon, both spoon levels are the same. (C) The inclination of the spoon indicates the posterior condylar axis when both spoons are inserted into the medial and lateral joint spaces.

In non-straight-type knees, the double-spoon technique is performed with custom-made accessory spoons (Figure 4, Panel A). The accessory spoon is 0.5 mm thick and inserted into the opposite joint space, then incorporated with the conventional spoon. The spoons are thus set at the same level (Figure 4, Panel B). When both spoons are inserted into both compartments, it indicates the PCA (Figure 4, Panel C). The coronal alignment of the cutting block is adjusted as the single-spoon technique.

**Figure 5:**
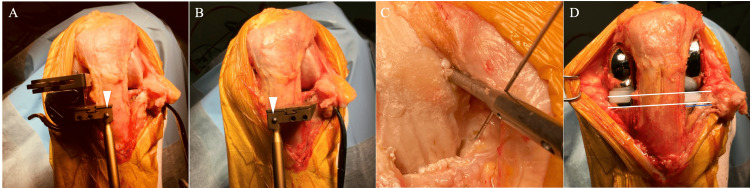
Bone-cutting procedure for a medial dominant osteoarthritis (A) After the inclination and level of the cutting block are decided, a headless pin (arrowhead) is inserted into the tibia through the most lateral pinhole of the cutting block. The medial procedure is then performed. (B) The medial cutting block is changed to the lateral cutting block using the same pin connected to the yoke, and the same extent, slide, and posterior slope are maintained. (C) When performing the horizontal cut, a 2 mm K-wire is inserted to prevent a horizontal overcut. (D) The lateral procedure is then performed to set the medial and lateral cutting lines to be parallel.

The spoon and the cutting block are fixed using the G-clamp, and the cutting block is fixed using a headless pin (Figure 5, Panel A). After the bone cut and adjustment of the flexion-extension gap are completed on the predominantly affected condyle, the cutting block is removed from the headless pin and changed to that on the opposite side using the same pin along with the extramedullary guide (Figure 5, Panel B) and the ankle yoke with retained extension, side-slide length, and posterior slope to maintain the cutting level as well as sagittal and coronal inclinations. The lesser affected compartment procedure is then performed. When the horizontal cuts are made, insertion of a K-wire at the tip of the tibial spine is recommended to prevent a horizontal overcut (Figure 5, Panel C). After the implantation, the CJL and cutting lines are virtually parallel (Figure 5, Panel D).

**Figure 6:**
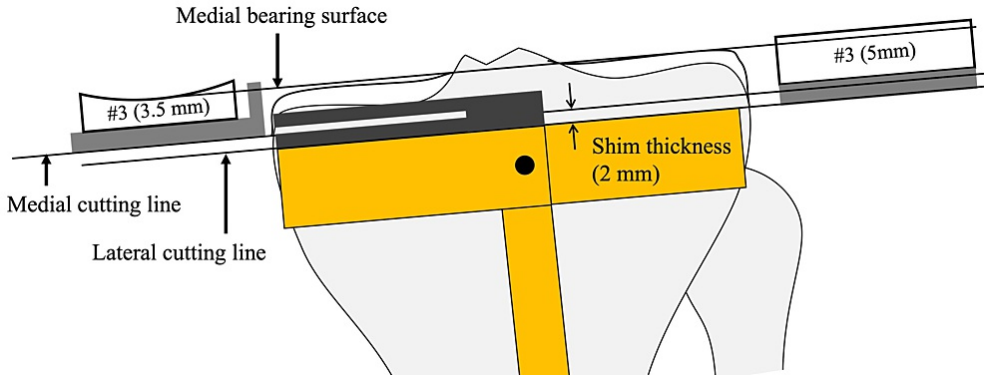
Medial and lateral tibial cutting level in the kinematic alignment Oxford unicompartmental knee arthroplasty The actual thicknesses are 0.5 mm and 2 mm thicker than the labeled number. Once the shim is removed, the lateral cutting level is 2.0 mm lower than that of the medial one. Eventually, the lateral bearing surface is 0.5 mm higher than the medial one when the same number bearing is used with the same cutting block level.

When the lateral tibial cuts are made, the shim is removed so that the cutting level is set to 2 mm lower than the medial cutting level. Both bearings are numbered, but the exact thicknesses of the bearings are 0.5 mm and 2.0 mm thicker than the labeled number. Complete leveling of both plateaus is therefore impossible when the level of the cutting block is constant (Figure 6). Eventually, the lateral CJL is inevitably 0.5 mm higher than the medial CJL, although this can be ignored.

Postoperative radiographical evaluation

**Figure 7:**
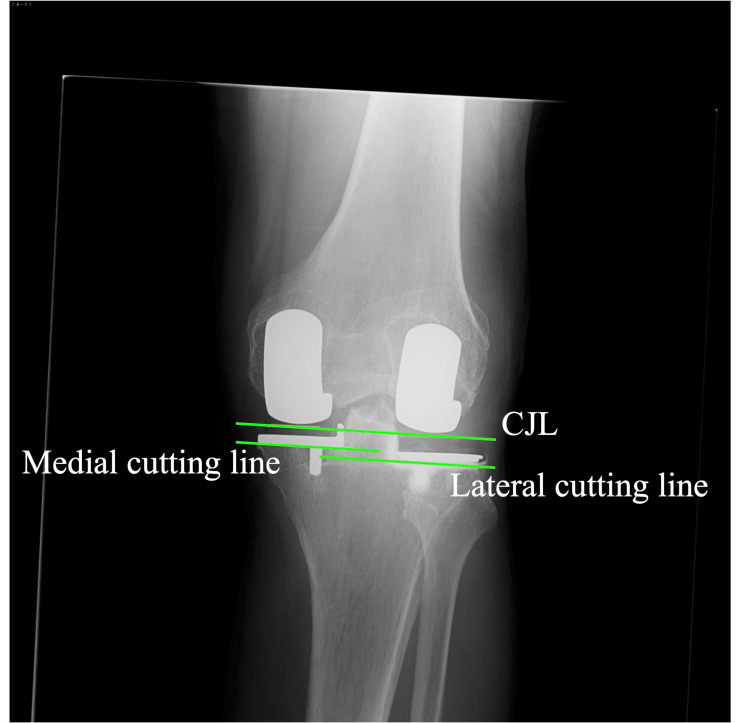
Postoperative radiography The coronal joint line (CJL) is parallel to the cutting lines. Note that the lateral cutting line is lower than the medial cutting line.

True anteroposterior radiography aligned to the tibial component surface is used for postoperative evaluation. Ideally, the postoperative CJL, which is the line tangential to both medial and lateral femoral components, is parallel with the medial and lateral cutting surface, and the medial cutting surface is 2 mm higher than the lateral cutting surface. In straight-type knees on the preoperative radiography, the CJL is expected to be parallel with the preoperative CJL (Figure 7).

## Discussion

This is the first report to document KA-BiUKA by OPK in detail. Robotic-assisted BiUKA using a fixed-bearing component was recently reported, and constitutional whole leg alignment and joint line obliquity were shown to be restored [19]. Regarding OPK, it was used for BiUKA in the initial stage of the OPK [15]. Pandit et al. reported the staged BiUKA - adding a lateral UKA after medial UKA due to lateral compartmental osteoarthritis and showed satisfactory results [16]. More recently, a gait analysis showed that the subjects with BiUKA using OPK had similar gait characteristics to the normal subject compared to TKA subjects [20]. BiUKA has been reported to have mechanical advantages. A compression force on one component would cause a lift-off of the other component in the one-piece TKA component, but it never occurs in the two-piece tibial components in BiUKA [15,21]. The bone-cutting line was not shown in the previous studies; however, it is thought to play an important role in load transmission. A slight varus implantation of the tibial component was reported in previous biomechanical studies to reduce stress concentration in the medial tibial cortex, but a valgus placement increases it [22]. Although avoidance of valgus placement is important, the placement can be valgus against the proximal tibia in knees with tibia vara, which is especially prevalent in Asian patients [23,24]. Component placement parallel to the CJL might enable a proportioned load transmission across the joint.

In our technique, the dominantly affected compartment is operated upon prior to the lesser affected compartment. In this sequence, the operated condyle is always normal or nearly normal. By contrast, the procedure of the lesser affected condyle can be influenced by the disease of the opposite condyle, such as contracted or relaxed soft tissue and cartilage as well as bone loss. Our technique is a tibia-first sequence, in contrast with most KA-TKA techniques, in which the femur-first technique is used [25,26]. However, this is a standard technique in OPK and has been used for more than 40 years [27]. The tibia-first approach and incremental gap adjustment using the milling system can facilitate easy and precise adjustment of flexion and extension gaps. We believe the dominantly affected condyle-first and the tibial-first sequence might be ideal for facilitating the KA-BiOUKA.

There are some limitations in our report and technique. First, it was necessary to use custom-made devices (accessory spoons and side-slidable ankle yoke). Although a similar operation can be performed without the custom-made devices, where the cutting levels are decided individually using the standard spoon, the cutting plane is not parallel to the CJL. The CJL could be made parallel to the original CJL, but the kinematics and load distribution might be affected. Second, the lateral component is set in varus in the technique. This alignment is equivalent to a valgus placement of the medial UKA, which has reportedly increased the mechanical stress on the tibial cortex. Therefore, it can increase the risk of failure. Varus placement has not been reported to increase the risk of failure in lateral UKA. Third, there was no evaluation of clinical outcomes, in particular its superiority over TKA. A larger number of cases and long-term studies are needed to prove the benefits of the KA-BiUKA. Lastly, we used medial and lateral capsulotomy and disturbance of blood supply for the patella, followed by the avascular necrosis of the patella, and the anterior knee pain is a concern. The medial parapatellar approach for femoral and tibial bone cuts like TKA along with small lateral capsulotomy for lateral gap evaluation using the feeler gauge might be helpful. However, it might require additional instruments.

BiUKA is a great technically demanding operation; therefore, the establishment of the procedure is necessary for a fair evaluation of its effect. Our technique is considered to be easy and reproducible, so it can be implemented widely.

## Conclusions

The details of an operative technique of BiUKA using OPK are presented as an alternative procedure for osteoarthritic knees with a functioning ACL and cartilage interaction on the lateral compartments. The technique can replicate the pre-arthritic joint line and maintain both cruciate ligaments, and a cylindrical axis is completely constructed. Moreover, the bone-cutting surface can be set in parallel to the joint line, which might be beneficial to load transmutation.
